# Improving Acute Orthopaedic Admission Note Documentation Standards at a Tertiary Centre Through Proforma Introduction: A Quality Improvement Initiative

**DOI:** 10.7759/cureus.30880

**Published:** 2022-10-30

**Authors:** Conor S O'Driscoll, Ross Condell, David O'Sullivan, Martin Davey, Stephen Kearns, Colin G Murphy

**Affiliations:** 1 Trauma and Orthopaedics, Galway University Hospitals, Galway, IRL; 2 Orthopaedics, Galway University Hospitals, Galway, IRL; 3 Trauma and Orthopaedics, Royal College of Surgeons, Dublin, IRL; 4 Orthopaedics, University Hospital Galway, Galway, IRL; 5 Trauma and Orthopaedics, Galway University Hospital, Galway, IRL

**Keywords:** patient safety, quality improvement, clinical handover, trauma and orthopedic surgery, documentation, admission proforma

## Abstract

Background

Clear, accurate written documentation plays an important role in the handover of medical information, helping to improve care efficiency and avoid medical errors. Both HSE and the Irish National Orthopaedic Models of Care guidelines include key documentation standards for admission notes. Standardised medical admission proforma can help improve documentation standards, but their usage across Irish orthopaedic units is limited to three centres. We evaluated whether an admission proforma improved the documentation standards of acute orthopaedic trauma admission notes within our regional trauma unit.

Methods

Cycle 1 consisted of a retrospective review of 50 consecutive acute orthopaedic trauma admissions. Exclusion criteria included planned admissions, age <16 years, spinal or pelvic trauma, and ‘hip’ fractures for whom an existing proforma was in use. Cycle 2 consisted of a prospective review of 50 consecutive acute trauma admissions using the new proforma. Each cycle was scored against a pre-determined checklist incorporating Irish Health Service Executive and Orthopaedic Models of Care documentation standards, with results collated and statistical analysis then performed using Fisher's exact test.

Findings

Significant improvements in admission note compliance with national documentation were observed. This encompassed multiple domains including clinical identification, e.g., consultant identification (78% to 100% p=0.0005), clinical history, time of injury (72% to 100% p=0.0001), medical history, smoking status (86% to 100% p=0.0001), and patient assessment, vital signs (28% to 70% p=0.0001).

Conclusion

Improvements in admission note compliance with national documentation standards followed the introduction of a standardised proforma. These findings may encourage the introduction of similar proforma in other units, with potential benefits in patient care.

## Introduction

From the moment of a patient's initial presentation, clear and accurate written documentation plays an important role in the handover of medical information, helping to improve care efficiency and avoid medical errors. This has only increased in importance over recent times due to the collaborative multidisciplinary nature of modern healthcare and the increasing adaptation of shift working in European Working Time Directive-compliant medical rosters [1,2], in the setting of increasing trauma presentations [3,4]. From a medicolegal perspective, comprehensive contemporaneous documentation provides an account of care provided to patients and is regularly referred to in legal proceedings [5]. The importance of maintaining high standards of written documentation is highlighted by the inclusion of key standards in both national and international guidance documents [6-8].

The admission note serves as a reference point throughout a patient's care journey for caregivers seeking patient information [9]. Standardised admission proforma has been demonstrated to be an effective tool to maintain high-quality documentation [9-11]. Proponents reason that they act both as an aide memoire for junior clinicians clerking patients' admissions while also improving legibility and aiding time efficiency [11]. Indeed, within the orthopaedic sphere, Jefferies et al. demonstrated that an admission proforma helped their Scottish trauma unit improve adherence to Royal College of Surgeons standards [12]. Despite this, regular admission notes proforma usage has not been widely adopted for general orthopaedic trauma admissions across Ireland, with a poll of 16 units performed as part of this study finding a current usage rate of only 18.8%.

Our study aim was to evaluate the effect of the admission note proforma introduction for acute orthopaedic trauma admissions and improve our adherence to key national guidelines. The general Irish Health Service Executive Standards and Recommended Practices for Healthcare Records Management and the HSE National Model of Care for Trauma and Orthopaedic Surgery documents were used as our project standards [7,8]. This information is useful to other trauma units and medical specialties considering similar admission proforma adoption.

## Materials and methods

This quality improvement project was performed with respect to Standards for Quality Improvement Reporting Excellence (SQUIRE) guidelines [13] at a regional trauma and tertiary referral centre in the West of Ireland [14]. First, a retrospective review of 50 consecutive unscripted admission notes was conducted independently by three authors, Conor O'Driscoll, Ross Condell, and David O'Sullivan. These notes were scored against a pre-determined checklist to assess compliance with national documents. This incorporated guidance regarding domains relating to the identification, the reason for presentation, background medical and social history, clinical assessment, care provided, and management plan. Inclusion criteria included adult patients admitted under the care of the orthopaedic team following acute traumatic injuries. Exclusion criteria included patients less than 16 years of age, secondary trauma admissions previously assessed at the clinic, proximal femoral ‘hip’ fractures, and spinal and pelvic trauma.

Following the dissemination of initial results at a weekly departmental teaching session, a standardised admission proforma document was produced in collaboration with multidisciplinary stakeholders. This incorporated recommendations from the key clinical guidelines and is attached in the appendix. Following a one-month introduction period, a further evaluation of 50 consecutive proforma admission notes was conducted using the same checklist. Throughout this period, on-call teams completing the documentation were not informed that their admission note was to be audited, so as to limit bias. Following the completion of this second cycle, the results from the review were collated using Microsoft Excel (Microsoft® Corp., Redmond, WA). Statistical analysis was performed through Fisher’s Exact Test using Stata software (StataCorp, College Station, TX). A p-value of <0.05 was used for statistical significance. Approval was granted by Galway University Hospital clinical audit board (Audit Number 225).

## Results

Significant improvements in admission note compliance with national documentation were observed across multiple clinical domains between the unscripted pre- and proforma post-intervention cycles. The results of each cycle are presented in Table 1.

**Table 1:** Results

	Unscripted cycle 1	Proforma cycle 2	Fishers exact test
Identification			
Patient details	100% (50/50)	100% (50/50)	1
Consultant on-call	78% (39/50)	100% (50/50)	0.0005
Admitting doctor’s full name	74% (37/50)	100% (50/50)	0.0001
Admitting doctor registration number	64% (32/50)	98% (49/50)	0.0001
Admitting doctor grade	92% (46/50)	82% (41/50)	0.0023
Date	94% (47/50)	98% (49/50)	0.62
Time	78% (39/50)	96% (48/50)	0.015
Clinical history			
Time of injury	72% (36/50)	100% (50/50)	0.0001
Mechanism of injury	92% (46/50)	100% (50/50)	0.12
Associated injury (presence/absence)	48% (24/50)	100% (50/50)	0.0001
Preceding/following symptoms	56% (28/50)	100% (50/50)	0.0001
Background history			
Medical conditions	100% (50/50)	100% (50/50)	1
Medications	86% (43/50)	92% (46/50)	0.52
Allergies	96% (48/50)	92% (46/50)	0.68
Smoking status	86% (43/50)	100% (50/50)	0.0001
Alcohol usage	42% (21/50)	94% (47/50)	0.0001
Hand dominance (upper limb trauma)	73% (19/26)	89% (25/28)	
Occupation	58% (29/50)	94% (47/50)	0.0001
Housing	62% (31/50)	94% (47/50)	0.0001
Patient assessment			
Vital signs	28% (14/50)	70% (35/50)	0.0001
Limb assessment	88% (44/50)	100% (50/50)	0.027
Neurological assessment	70% (35/50)	98% (49/50)	0.0002
Vascular assessment	72% (36/50)	96% (48/50)	0.0019
Other systems	14% (7/50)	74% (37/50)	0.0001
Management			
Investigation/treatments to date	78% (38/50)	98% (49/50)	0.0018
Management plan	100% (50/50)	100% (50/50)	1

## Discussion

The results of our study demonstrate the considerable improvements in documentation standards that can be achieved through the introduction of an admission proforma document. This has been well received across the department, with junior clinicians citing benefits in time efficiency and as an aide memoire. Nursing and multidisciplinary colleagues also credit the proforma with improved legibility and ease of information retrieval.

Clear identification of responsible clinicians for each patient is a cornerstone of good clinical practice, streamlining communication during urgent care requirements and ensuring accountability. [15] Improvements in consistency between cycles of this audit are beneficial in this regard. Clear documentation of time and date allows the clinical course to be monitored and a timeline of changes in clinical status to be established [16]. They also allow for the timing of previous interventions to be ascertained, thus enabling informed future decision-making [17].

A high proportion of routine trauma admissions are for patients in the geriatric age categories over 65. Comprehensive assessment and documentation of preceding and following symptoms may help identify possible causative factors relating to other systems, such as syncope or dizziness [18]. Clear demonstration of these factors on the admission note helps guide appropriate further investigation [19]. It can also help ensure the identification of medical comorbidities that can be optimised as early as possible, limiting anaesthetic surgical delays [20]. In addition, so-called "silver trauma" of patients greater than 65 years of age is commonly associated with multiple associated injuries which are often overlooked, with delayed recognition and specialist care associated with poorer outcomes as identified by a recent UK-wide trauma audit report [21]. The placement of this section on the proforma reminds clinicians to comprehensively assess patients for the same, with a significant improvement in levels of documentation regarding both clinical symptoms and associated injuries seen in our study.

Improved documentation of patient living situations helped alert multidisciplinary team members to identify potential barriers to discharge such as isolation and multistory homes early in a patient's stay. This helped in appropriate allocation of resources, with targeted packages such as homecare packages being organised early. This helped maintain patient flow and has been identified across the literature as being beneficial to patient outcomes [22]. Likewise, screening for alcohol and smoking status improved across our study, facilitating onward referral to specialist liaison services.

An analysis of 8548 trauma and orthopaedic litigation and medicolegal claims in the United Kingdom over a ten-year period found a significant amount of damages paid for burns and bruising, neurological injuries, and damage to limbs [23]. Meticulous documentation of a comprehensive physical examination on initial presentation is crucial in the defence of such cases, allowing the timing of these injuries to be established. The clear separation of sections for specific and general components in the proforma helped to improve compliance in our unit.

Regarding potential limitations, as is a feature of all such studies, bias may have been introduced through the retrospective nature of the study. As part of the intervention, an education session accompanied the proforma introduction, which may have contributed partly to the significant improvement seen, as seen in other audit studies [24]. Nonetheless, we feel that these findings are of benefit to the broader orthopaedic community, providing encouragement and a roadmap for the introduction of admission proforma for acute orthopaedic trauma admission in other units which will only be of benefit to patient care.

## Conclusions

Significant improvements in admission note documentation standards across multiple domains were observed following the standardised proforma's introduction in our orthopaedic unit. This helped to streamline communication between multidisciplinary team members, with resultant benefits in patient care and healthcare efficiency. The proforma introduction also ensured documentation of a comprehensive patient assessment, which aids in the identification of associated injuries and is beneficial in the defence of potential medicolegal claims. The findings of this study may encourage the introduction of similar proforma in other units.
